# Pharmacological Application of Growth Factors: Basic and Clinical

**DOI:** 10.1155/2015/141794

**Published:** 2015-01-05

**Authors:** Jian Xiao, Yadong Wang, Saverio Bellusci, Xiaokun Li

**Affiliations:** ^1^School of Pharmacy, Wenzhou Medical University, Chashan University-Town, Wenzhou 325035, China; ^2^Department of Bioengineering, McGowan Institute for Regenerative Medicine, University of Pittsburgh, Pittsburgh, PA 15261, USA; ^3^Excellence Cluster Cardio Pulmonary System, University Justus Liebig Giessen, Aulweg 130, 35392 Giessen, Germany

Growth factors are signaling molecules that are typically secreted at the site of repair by many different cell types including platelets, stem cells, and fibroblasts. Since Montalcini and Hamburger first described the nerve growth factor (NGF) in 1951, this epoch-making discovery has initiated the detection of a multitude of other growth factors that alter cell growth, cell differentiation, and proliferation, which is essential for wound healing and tissue repair and regeneration. Several growth factors such as fibroblast growth factor (FGF), epidermal growth factor (EGF), and nerve growth factor (NGF) play a key role in brain or spinal cord injury and acute organ injuries. Vascular endothelial growth factor (VEGF) increases vascular permeability, induces angiogenesis, vasculogenesis, which is applied in treatment of ischemic heart disease.

Treatment with growth factors is beginning to gain worldwide prevalence, mainly in plastic and reconstructive surgery. However, the molecular mechanisms of growth factors treatment are still undefined. Thus, further investigations on mechanisms of growth factors in basic and clinical research are urgent. Therefore, we have invited the researchers to contribute few research/review papers to provide evidence that supports the application of growth factor in prevention or treatment of diseases.

In this special issue, we have invited some papers hoping to shed light on some aspects of this very interesting field. We have collected 8 papers by scientists from 4 countries. In the submitted research papers, H. Wang et al. summarize the current understanding of the NGF signaling in retina and the therapeutic implications in the treatment of glaucoma. NGF offers the promise of actually restoring visual function through acting on the TrkA receptor; however, the future of NGF-dependent treatments in the armamentarium of glaucoma therapy as most of the present studies were in animal models, hence, randomized, controlled glaucoma clinical trials need to be performed to evaluate the therapeutic effect of NGF in the treatment of glaucoma. While M. Ammendola et al. review antitumor and antiangiogenic potential of three agents which are able to inhibit the functions of mast cells (MCs) tryptase: gabexate mesylate, nafamostat mesylate, and tranilast, the authors suggest that future awaited clinical studies aim to evaluate the truly efficacy of the tryptase inhibitors as a novel tumor antiangiogenic therapy. J. Cai et al. concluded the neuroprotective efficacy of neurotrophins (NTs) (NGF, BDNF, FGF-2, IGF, NT3, and NT4/5) in animal models, highlighted outstanding technical challenges, and discussed more recent attempts to harness the neuroprotective capacity of endogenous NTs using small molecule inducers and cell transplantation. On the other hand, J.-C. Chen and colleagues demonstrated that NGF exist multiple bioactivity except for the neuronprotective activity. They found NGF accelerates the healing of skin excisional wounds in rats and the fibroblast migration induced by NGF may contribute to this healing process; moreover, the activation of PI3K/Akt, Rac1, JNK, and ERK may be involved in the regulation of NGF-induced fibroblast migration. In two very interesting research papers, Z.-G. Feng et al. have shown that tobacco plants express Keratinocyte Growth Factor (KGF1) via Agrobacterium-mediated transformation using a Potato virus X- (PVX-) based vector (pgR107). The plant-derived KGF1 promotes the proliferation of NIH/3T3 cells and significantly stimulates wound healing in the diabetic wounded rat model. This finding indicated that KGF1 from tobacco maintains its biological activity, implying prospective industrial production in a plant bioreactor. While X.S Wang suggested endoplasmic reticulum (ER) stress is the key mechanism for regulating FGF21 in several metabolic diseases. This study showed FGF21 is the target gene for activating transcription factor 4 (ATF4) and CCAAT enhancer binding protein homologous protein (CHOP). ER stress increased the half-life of mRNA of FGF21, which may partly explain the mechanism of increasing FGF21 levels in metabolism disease. In the following papers, H. Nawa et al. discussed neuregulin-1 (NGR1) and EGF to rodent pups, juveniles, and adults and characterized neurobiological and behavioral consequences. The cytokine-driven dopaminergic dysfunction might illustrate some of the psychopathological features of schizophrenia, although it is possible that the responsible factors might be other cytokines other than EGF, NRG1, or virokine. L.-J. Xiang ea al. investigated the hair growth promoting activities of three approved growth factor drugs, FGF-10, FGF-1, and FGF-2. They observed that FGFs promoted hair growth by inducing the anagen phase in telogenic C57BL/6 mice. FGFs-treated group showed earlier induction of *β*-catenin and Sonic hedgehog (Shh) in hair follicles, suggesting that FGFs promotes hair growth by inducing the anagen phase in resting hair follicles and might be a potential hair growth-promoting agent. Finally, J. Song et al. summarized the recent findings on the association between risk factors for vascular dementia and adiponectin including aging, diabetes, hypertension, atherosclerosis, and stroke. The authors suggested that further studies are necessary to examine the role of adiponectin in vascular dementia, and the regulation of adiponectin levels and receptors of adiponectin would be important for the prevention and treatment of vascular dementia.

